# Perception of the Accreditation of the National Commission for Academic Accreditation and Assessment at Different Health Colleges in Jeddah, Saudi Arabia

**DOI:** 10.7759/cureus.43871

**Published:** 2023-08-21

**Authors:** Ali S Al-Shareef, Mansour A AlQurashi, Azza Al Jabarti, Hend Alnajjar, Ahmad A Alanazi, Mohamed Almoamary, Bader Shirah, Khalid Alqarni

**Affiliations:** 1 Department of Emergency Medicine, King Abdulaziz Medical City, Jeddah, SAU; 2 Research Office, King Abdullah International Medical Research Center, Jeddah, SAU; 3 College of Medicine, King Saud bin Abdulaziz University for Health Sciences, Jeddah, SAU; 4 Department of Pediatrics, Neonatology Division, King Abdulaziz Medical City, Ministry of National Guard Health Affairs, Western Region, Jeddah, SAU; 5 College of Nursing, King Saud bin Abdulaziz University for Health Sciences, Jeddah, SAU; 6 College of Applied Medical Sciences, King Saud bin Abdulaziz University for Health Sciences, Riyadh, SAU; 7 Research Office, King Abdullah International Medical Research Center, Riyadh, SAU; 8 Department of Medicine, King Abdulaziz Medical City, Riyadh, SAU; 9 College of Medicine, King Saud bin Abdulaziz University for Health Sciences, Riyadh, SAU; 10 Department of Neuroscience, King Faisal Specialist Hospital & Research Centre, Jeddah, SAU; 11 College of Applied Medical Sciences, King Saud bin Abdulaziz University for Health Sciences, Jeddah, SAU

**Keywords:** saudi arabia, perception, ncaaa, healthcare, college, university, accreditation

## Abstract

Introduction

Following the guidelines for maintaining quality set forth by the National Commission for Academic Accreditation and Assessment (NCAAA) accreditation procedure, Saudi higher education institutions, including health sciences colleges, must adhere to these guidelines. This study aims to assess the perception of personnel involved in NCAAA accreditation processes about the purpose, process, motivation, and level of involvement in the NCAAA accreditation at King Saud bin Abdulaziz University for Health Sciences (KSAU-HS).

Methods

The study was conducted at KSAU-HS, Jeddah. The participants included 15 administrators and 32 faculties from the College of Medicine, College of Applied Medical Sciences, and College of Nursing with experience in the NCAAA process. A questionnaire was used to determine how motivated and involved people feel about the accreditation process. Data were examined statistically with SPSS (Version 23; IBM Corp., Armonk, NY, USA), and descriptive statistics were used.

Results

Forty-seven participants (23 men, 24 women, ages 36 to 55) took part in the study, of which 68% were faculty members and 32% were administrators with a variety of skill sets from the three colleges. Most participants displayed a positive attitude toward the NCAAA accreditation's motive and level of commitment.

Conclusions

Most of the participants in the current study contended with the NCAAA process and deemed it substantial long-term improvements.

## Introduction

Saudi Arabia is currently beholding progress in all human development indices including healthcare services and education and has the potential for comprehensive development. This is a reflection that the country has enjoyed tangible economic prosperity, and the best is yet to come. With the rapid growth in education generally and health education particularly, the demand emerged for a national entity for quality assurance. Therefore, the National Commission for Academic Accreditation and Assessment (NCAAA) was established for monitoring and improving the quality of public and private higher education institutions and programs [1]. The NCAAA is an independent organization that has developed guidelines and criteria for a systematic accreditation process. The Ministry of Education urged all higher education institutions, including health colleges to maintain and assure quality that satisfies the NCAAA accreditation process [2]. Undergraduate medical education requires accreditation to assess the contributions of institutions to adopt a culture of quality in medical institutions [3].

Accreditation is the process of evaluating educational institutions based on established criteria, standards, and procedures by external regulatory bodies appointed by government entities. It involves collecting data about multiple aspects of the educational institution and making decisions about compliance with the standards. It is undertaken for several primary reasons, including to ensure quality education and guarantee equal standards for medical school graduates, as well as to establish the minimum essential requirements for every medical school to meet. The majority of accreditation processes are indistinguishable from processes applied to any higher education institution, but some are distinct to medical colleges [4].

The Education Training Evaluation Commission (ETEC) Center aims to improve the quality and excellence of higher education institutions and programs through accreditation and academic evaluation. Under the Center's umbrella, one of the strategic initiatives is to improve and streamline the accreditation process, including identifying and simplifying the review process. Additionally, the academic team and the administrative assistant provide positive support for these efforts [5]. There are several steps involved in the accreditation process. The requirements of accreditation vary depending on the type of accreditation that is being pursued, but the process is similar for all types of accreditation [6]. The fundamental key steps for the accreditation process started with self-assessment and ended with successful accreditation [7]. All of the steps of the accreditation process can be achieved only by a team that knows the value of accreditation and admits this issue in all tasks provided by them [8].

Due to the relatively recent implementation of accreditation in Saudi Arabia, several aspects remain unknown, such as the perceptions of responsible personnel to implement the accreditation (e.g., faculty members and administrators) are unknown. The literature is scant on studies that focus on the perception of people who are involved in the accreditation process in Saudi Arabia. For example, Alasker examined the perceptions of 189 faculty members and administrators about several aspects related to accreditation, including the process, purpose, motivation, and level of involvement in the accreditation process. It was found that the involvement of those with teaching experience and the accreditation process were positively affected. The need for improving awareness regarding the accreditation process and establishing a quality culture with support from the administration was highlighted by the participants in the study [9].

Determining the perception and motivation of faculty members and administrators who are involved in implementing accreditation is significant in anticipating the success of the process of accreditation. In this study, we aimed to evaluate the perception of personnel involved in the NCAAA accreditation process about the purpose, process, motivation, and level of involvement in the NCAAA accreditation at different colleges of King Saud bin Abdulaziz University for Health Sciences (KSAU-HS), Jeddah, Saudi Arabia. The main rationale of the study was to enhance the involvement of faculty and the authorities concerned by knowing the areas of lacunae where they are not contended and further improvise the standards of the educational institution.

## Materials and methods

This is a cross-sectional observational descriptive study that was conducted at KSAU-HS, which is a government university specializing in health sciences. KSAU-HS is accredited by the NCAAA/ETEC and supervised by the Ministry of Education in the Kingdom of Saudi Arabia for its various programs for undergraduate and postgraduate degrees. The study was conducted at the Jeddah branch of KSAU-HS. All colleges that had experience with the NCAAA accreditation (i.e., College of Medicine [COM], College of Nursing [CON], and College of Applied Medical Sciences [CAMS]) were included. All faculties and administrators working in the previous settings and fulfill the inclusion criteria were recruited (a total of 47 individuals including 15 administrators and 32 faculties). Convenience sampling technique was used in the recruitment of a purposeful sample of those involved in the NCAAA accreditation process. One tool was used to collect the necessary data in the current study. This study utilized the questionnaire that was developed and validated by Alaskar [9]. The questionnaire consisted of nine demographic items and 34 on a five-point Likert scale with a range of answer options that go from strongly agree to strongly disagree. Additionally, the questionnaire included seven questions about the variable perception of purpose, 10 questions about the perception of the process, eight questions about motivation, and nine questions about the level of involvement.

The Institutional Review Board approval (IRBC/0956/21) from King Abdullah International Medical Research Center was obtained prior to initiating the study. Permission to use the questionnaire was obtained. The content validity of the study tool was tested by Alaskar [9] and the reliability of the tool was tested also using a Cronbach’s alpha coefficient test where the Cronbach’s alpha ranged from 0.7384 to 0.8617, which indicates a high level of internal consistency for the scale. To reach the participants (faculties and staff who work in the targeted colleges), the questionnaire alongside the informed consent was prepared electronically on Google Forms (Google LLC, Mountain View, CA, USA), and the links were e-mailed to the targeted participants. Instructions on how to complete the questionnaire were explained on the first page of the electronic questionnaire. Participation was voluntary, and answers were anonymous. Numerical values and demographic data were calculated and presented using frequency and percentage. Data were analyzed using IBM Statistical Package for the Social Sciences (SPSS) version 23 (IBM Corporation, Armonk, NY, USA).

## Results

The response of 47 individuals were included in the analysis as per the inclusion criteria. There were 24 female and 23 male participants and the majority were in the age range of 36-55 years (77%). Faculty members represented 68% of the participants, while administrators represented 32%. Approximately 75% of the participants worked more than five years at their colleges. Most participants (83%) had higher education qualifications. Approximately half of the participants had previous experience with the NCAAA accreditation. Most of the participants were from the following colleges: COM (49%), CAMS (32%), and CON (19%) (Table 1).

**Table 1:** Distribution of the study subjects according to their demographic profile (N = 47)

Demographic profile of the participants	Frequency (N = 47)	%
Age (Years)		
20-35	9	19
36-45	21	45
46-55	15	32
56-65	2	4
Gender		
Male	23	49
Female	24	51
Role within the college/deanship		
Administrator	15	32
Faculty	32	68
Number of years teaching/working		
Less than 1 year	0	0
1-5 years	12	26
6-10 years	22	47
Over 10 years	13	28
Level of education		
Diploma	0	0
Bachelor	5	11
Master	16	34
PhD	23	49
Other	3	6
Experience of any form of academic accreditation		
Yes	26	55
No	21	45
College		
College of Medicine	23	49
College of Nursing	9	19
College of Applied Medical Sciences	15	32

Most participants had a positive response (either agree or strongly agree) to most of the items in the questions about the perception of the purpose of NCAAA accreditation. For example, 85% of the participants agreed and strongly agreed that the NCAAA accreditation provides an effective national system for assuring quality in higher education while 81% of them agreed and strongly agreed that the NCAAA program accreditation provides an important process for improving the quality of medical and health academic programs. More than half of the respondents strongly disagree and disagree with the statement that shows the NCAAA program accreditation does not provide assurance that programs meet established quality standards. Eighty-one percent of the participants strongly agreed and agreed that it is effective to distinguish between the purpose of institutional and program accreditation. Of the total participants, 66% believed that peer evaluation is a major strength of the program accreditation while only 26% strongly agreed and agreed that graduation from accredited programs is not required for being licensed in the medical and health professions. Seventy-five percent of the participants strongly agreed and agreed that the program benefits from periodic self-evaluation required by the accrediting agency (Table 2).

**Table 2:** Distribution of the study subjects according to their perception of the purpose of National Commission for Academic Accreditation and Assessment (NCAAA) accreditation (N = 47)

Statements	Strongly Disagree		Disagree		Neutral		Agree		Strongly Agree		Don’t know / Doesn’t apply to me	
	N	%	N	%	N	%	N	%	N	%	N	%
NCAAA accreditation provides an effective national system for assuring quality in higher education.	2	4	0	0	1	2	10	21	30	64	4	9
It is effective to distinguish between the purpose of institutional and program accreditation	2	4	0	0	2	4	17	36	21	45	5	11
NCAAA program accreditation provides an important process for improving the quality of medical and health academic programs	2	4	0	0	2	4	9	19	29	62	5	11
NCAAA program accreditation does not provide assurance that programs meet established quality standards	6	12.5	19	40	12	26	4	9	6	12.5	0	0
Peer evaluation is a major strength of the program accreditation	1	2	1	2	6	13	14	30	17	36	8	17
Graduation from accredited programs is not required for being licensed in the medical and health professions	14	30	5	10.5	5	10.5	8	17	9	19	6	13
The program benefits from periodic self-evaluation required by the accrediting agency	2	4	0	0	3	6	13	28	22	47	7	15

Regarding participants’ response to the process of NCAAA accreditation, 73% agreed and strongly agreed that self-study is an effective component of the accreditation process, while 75% of them agreed and strongly agreed that the evaluation of the program’s self-study by peer evaluators against the standards is an effective feature of the accreditation. Most of the participants agreed that the primary purpose of the site visit is to evaluate compliance with the program practices and identify areas of improvement. Forty-two percent disagreed and strongly disagreed that the program accreditation does not need to be concerned with inputs and processes that lead toward the program's effectiveness. Fifty-seven percent agreed and strongly agreed that the program accreditation has shifted its emphasis from process to outcomes results. That the program accreditation process was found to stimulate long-term improvements was agreed upon by 72% of the participants (Table 3).

**Table 3:** Distribution of the study subjects according to their perception of the process of National Commission for Academic Accreditation and Assessment (NCAAA) accreditation (N = 47)

Statements	Strongly Disagree		Disagree		Neutral		Agree		Strongly Agree		Don’t know / Doesn’t apply to me	
	N	%	N	%	N	%	N	%	N	%	N	%
The self-study is an effective feature of the accreditation	1	2	1	2	3	6	14	30	20	43	8	17
Evaluation of the program’s self-study against standards /essentials by a site visit team of peer evaluators is an effective feature of the accreditation	1	2	3	6	3	6	16	34	19	41	5	11
The primary purpose of the site visit is to evaluate compliance of the program practices with published criteria of standards/essentials	1	2	2	4	2	4	14	30	22	47	6	13
The primary purpose of the site visit is to identify areas of improvement	2	4	2	4	4	9	14	30	19	40	6	13
Site visit teams are typically composed of peers from other programs with similar missions	1	2	1	2	6	13	19	41	11	23	9	19
Selection of peer evaluators for a site visit team is made primarily by professional staff of the accrediting agency	0	0	1	2	4	9	14	30	17	36	11	23
Selection of peer evaluators for a site visit team is a shared decision among the accrediting agency, program director and visiting team	4	9	3	6	5	11	15	32	13	27	7	15
The program accreditation has shifted its emphasis from process to outcomes results	0	0	5	11	5	11	14	30	13	27	10	21
The program accreditation does not need to be concerned with inputs and processes that lead toward the program effectiveness	8	17	12	25	8	17	8	17	6	13	5	11
The program accreditation process stimulates long-term improvements	2	4	0	0	3	7	9	19	25	53	8	17

Nearly 30% of the participants agreed and 21% strongly agreed that they mainly conduct self-study activities because of the accreditation requirements. Most of the participants agreed that the improvement of the program and services is the primary motivation for academic accreditation on their campus. Forty-eight percent agreed and strongly agreed that if there were no outside requirements or mandates, their commitment to academic accreditation activities would probably diminish. Half of the participants disagreed and strongly disagreed that the academic accreditation does little to affect the true quality at our institution, while only 28% agreed and strongly agreed that the academic accreditation had no effect on the quality of their institution. The following statement was chosen almost equally by the participants who agreed (33%) and disagreed (32%) that ‘changes happen so slowly at their institution based on particular evaluations’. Of the total participants, 34% disagreed that their institution offers such quality that academic accreditation initiatives can do little to improve it, while 37% agreed with that statement (Table 4).

**Table 4:** Distribution of the study subjects according to their perception of the motivation toward National Commission for Academic Accreditation and Assessment (NCAAA) accreditation (N = 47)

Statements	Strongly Disagree		Disagree		Neutral		Agree		Strongly Agree		Don’t know / Doesn’t apply to me	
	N	%	N	%	N	%	N	%	N	%	N	%
We mainly conduct self-study activity because of the accreditation requirements	1	2	8	17	9	19	14	30	10	21	5	11
Improvement of the program and services is the primary motivation for academic accreditation in our campus	2	4	2	4	3	7	11	24	21	46	7	15
If there were no outside requirements or mandates, our commitment to academic accreditation activities would probably diminish	0	0	12	26	7	15	13	28	9	20	5	11
Evaluating the effectiveness of our accreditation process is a natural extension of the ideals of investigation and inquiry within the college	1	2	2	4	6	13	15	33	15	33	7	15
Academic accreditation does little to affect the true quality at our institution	11	24	12	26	7	15	7	15	6	13	3	7
Changes happen so slowly at our institution that it’s hard to specify what changes are based on particular evaluations	5	11	9	19.5	13	28	9	19.5	6	13	4	9
The results of our academic accreditation process seem to be more important to outside stakeholders than to our campus community	9	19	12	26	5	11	10	22	7	15	3	7
Our institution offers such quality that academic accreditation initiatives can do little to improve it	8	17	8	17	10	22	7	15	10	22	3	7

Only 52% of the respondents participated in reviewing their institution’s mission statement, while 67% of them were highly involved in academic accreditation activities at their institution. Sixty percent helped formulate assessment techniques to measure progress towards area goals in their programs. Of the total participants, 68% disagreed and strongly disagreed with the following statement ‘I am not familiar with the academic accreditation plan for my area’, and 74% of them improved academic skills because of specific assessment results from their academic accreditation activities. Surprisingly, 20% of the participants agreed with and 15% were neutral about the following statement ‘I am not personally aware of the benefits of academic accreditation activities’ (Table 5).

**Table 5:** Distribution of the study subjects according to their perception of the level of involvement in the National Commission for Academic Accreditation and Assessment (NCAAA) accreditation (N = 47)

Level of Involvement	Strongly Disagree		Disagree		Neutral		Agree		Strongly Agree		Don’t know / Doesn’t apply to me	
	N	%	N	%	N	%	N	%	N	%	N	%
I have participated in a review of my institution’s mission statement	8	17	3	7	6	13	9	19	15	33	5	11
I am highly involved in academic accreditation activities at my institution	2	4	3	7	5	11	11	24	20	43	5	11
I have participated in defining specific goals for my area	2	4	4	9	4	9	12	26	19	41	5	11
I have helped formulate assessment techniques to measure progress towards area goals	2	4	3	7	8	18	14	30	14	30	5	11
I am not familiar with the academic accreditation plan for my area	20	44	11	24	5	11	2	4	7	15	1	2
I have engaged in specific assessment exercises to aid my institutions in academic accreditation activities	1	2	1	2	5	11	12	26	21	46	6	13
I have made improvements because of specific assessment results from our academic accreditation activities	1	2	1	2	5	11	17	37	17	37	5	11
I am not personally aware of the benefits of academic accreditation activities	21	46	7	15	7	15	4	9	5	11	2	4
We should use the results of academic accreditation activities to support budget requests	2	4	2	4	4	9	9	20	22	48	7	15

## Discussion

The major goal of academic accreditation is to improve the quality of public and private institutions including health colleges which consequently assist in preparing well-equipped students and future healthcare providers for the needs and expectations of the public and adjusting to the rapid changes in healthcare service systems. The accreditation process ensures the minimum important requirements that each health college should provide [10]. Academic accreditation evaluates academic institutions and programs against pre-established standards by trained peer reviewers [11]. Faculty members and administrators are the major players in the process of accreditation who apply those standards. Therefore, it is necessary to explore their perception and motivation about the accreditation. Our study examined the perception of personnel involved in the NCAAA accreditation process at KSAU-HS in Jeddah, Saudi Arabia. The present data of 47 respondents showed that most of them were faculty members (68%), while the remaining were administrators from three colleges. Most of them were from the COM and had six to 10 years of working experience.

Most of the participants believed that the NCAAA accreditation provides an effective national system for assuring quality in higher education and an important process for improving the quality of healthy academic programs. Baker et al. [12] surveyed 595 deans and program directors of different educational institutions offering several allied health specialties (e.g., clinical laboratory sciences, physical therapy, occupation therapy, medical technology) about the importance of academic accreditation in four areas: the process, purpose, effectiveness, and critique and reform. The overall results depicted that the respondents confirmed the value of the accreditation to improve quality in higher education and showed support to all the surveyed areas.

Of the total participants in this study, 66% believed that peer evaluation is a major strength of the program accreditation, and 75% of them agreed that the evaluation of the program’s self-study by peer evaluators against the standards is an effective feature of the accreditation. In Baker et al.'s study [12], the deans and program directors were against the government standards and peer evaluation. The type and number of standards, cost, effort, and coordination may be the reason for this rejection. Therefore, the authors stressed the need for a greater understanding of the process and participation. In the current study, only 26% strongly agreed and agreed that graduation from accredited programs is not required for being licensed in the health professions. The higher competition in the job market made employers preferably hire graduates from accredited institutions because this determines the validity of programs of study and whether a graduate is qualified [13]. Almost 75% of the participants agreed that the program benefits from periodic self-evaluation required by the accrediting agency. Generally, the accreditation process leads to quality improvements [4].

The perceptions of process mainly investigated the faculty members' and administrators’ understanding of the importance of site visits and whether the published standards were met. The participants in our study agreed that the primary purpose of the site visit is to evaluate compliance with the program practices identifies areas of improvement. In educational institutions, the accreditation process provides a benchmark of education including teaching, research, and community services by which these institutions can be measured and compared to reach for excellence. General accreditation programs resulted in the improvement of the structure, process of care, and clinical outcomes in health centers [11]. The benefits of the accreditation process in educational institutions extend to provide a continuous process of quality improvement of the curriculum [14,15]. Al Mohaimeed et al. [4] found that the accreditation led to major changes in education processes and administration of curriculum and improved the quality of education.

Although most of the participants agreed that improvement of the program and services is the primary motivation for academic accreditation in their campuses, half of them would not be committed to quality improvement including self-study activities if there were no outside requirements by the NCAAA. The perception of motivation and positive attitudes toward accreditation improved management and quality of care. A review of 17 studies that focused on exploring healthcare professionals’ attitudes toward accreditation found the majority endorsed accreditation. The review’s findings depicted that 77% of the teaching hospital staff supported the preparation for accreditation as an important stage for developing the healthcare settings. Negative attitudes can be also related the accreditation, such as cost, time, and effort [11]. Accreditation is viewed by some faculty members and administrators as a distraction from their administrative roles [16].

Most of the participants in our study were highly involved in academic accreditation activities at their colleges. However, the limited awareness of some of the participants regarding the benefits of academic accreditation activities may be related to the fact that accreditation is a new concept among faculty members and administrators in the Kingdom [9]. All teaching staff should be involved in the process of self-assessment and improvements through their contributions and involvement in all activities. Faculty involvement and support are critical to quality improvement and successful accreditation [13].

The study has a few limitations. First, the number of participants was limited maybe because the inclusion criteria were limited to the colleges in one city. Second, the authors did not control for independent variables, such as age, gender, academic ranking, years of experience, college name, and role within the college. Finally, the used survey may not have been sensitive enough to capture all the participants’ characteristics and their perceptions and motivation

Based on the findings of this study, several recommendations are suggested. Although the participants in our study recognized the importance of accreditation in the quality of education and its outcomes, their involvement should be increased and encouraged. The commitment to quality starts at the highest level of the educational institutions so the leadership to establish an effective quality culture should be emphasized. A well-planned and structured educational program should be undertaken to improve the level of awareness and contribute to better practice toward accreditation by each faculty and administrator. Conducting accreditation requirements awareness campaigns based on the pre-assessment of faculty and administrator weaknesses and strengths considering their needs using the issued NCAAA guidelines is advised. Using mass media to broadcast all relevant, culturally acceptable messages regarding accreditation using weekly tips via email or other communication systems in the institution. Future studies could investigate the correlation between the perception of the purpose, process, and motivation towards NCAAA accreditation, and the perception of the level of involvement in NCAAA accreditation.

The findings of the current study have implications for practice. The information included in this study can be used to inform accreditation agencies and administrators about the importance of understanding how to motivate faculty to become more involved in the accreditation process. According to the findings of this study, administrators and accreditation agencies should consider improving the knowledge and understanding of staff involved in the accreditation process. Effective accreditation requires additional training and engaging faculty considering a "No blame" culture that encourages a positive attitude towards accreditation. Faculties and administrators should focus on the creation, cultivation, sustaining, and measuring of student learning outcomes of the curriculum. An accreditation strategy for upcoming accreditations and re-designations must be developed and revised continuously. Increasing faculty involvement in self-study activities of their institution must be appreciated. Highlighting the achievements of all who are involved in accreditation must be encouraged.

## Conclusions

Based on the findings of the current study, it can be concluded that accreditation is vital for meeting quality standards and encouraging continuous improvement. The NCAAA has played an important role in enhancing the quality of practice in higher education institutions in Saudi Arabia. The findings of this study contribute to the shortage of data regarding faculty members’ and administrators’ perceptions and motivation about the academic accreditation process at Saudi health colleges which may significantly change the practices of quality in these colleges.

## References

[1] (2021). About NCAAA: Establishment. https://www.ncaaa.org.sa/enportal/aboutcenter/pages/default.aspx.

[2] (2021). Standards for Quality Assurance and Accreditation of Higher Education Institutions. https://etec.gov.sa/en/productsandservices/NCAAA/Accreditation/Documents/D.1.I_%20Standards%20for%20Institutions_%20Final%202013.pdf.

[3] Al-Shehri AM, Al-Alwan I (2013). Accreditation and culture of quality in medical schools in Saudi Arabia. Med Teach.

[4] Al Mohaimeed A, Midhet F, Barrimah I, Saleh MN (2012). Academic accreditation process: experience of a medical college in saudi arabia. Int J Health Sci (Qassim).

[5] (2021). The Education Training Evaluation Commission (ETEC) Center. Steps for Program Accreditation. https://etec.gov.sa/en/productsandservices/NCAAA/AccreditationProgrammatic/Pages/StepsofAccreditation.aspx.

[6] 6] Standards Council of Canada. (2020 (2021). Standards Council of Canada. Steps to Accreditation. https://www.scc.ca/en/accreditation/get-accredited/steps.

[7] 7] Commission on Accreditation of Ambulance Services (CAAS). (2018 (2021). Commission on Accreditation of Ambulance Services (CAAS). https://www.caas.org/about/five-steps-to-accreditation/.

[8] (2021). AACSB Accreditation. https://www.aacsb.edu/educators/accreditation.

[9] Alaskar AA (2018). Accreditation perceptions and involvement in Saudi Arabian schools of nursing [dissertation]. https://scholarsrepository.llu.edu/cgi/viewcontent.cgi?article=1518&context=etd.

[10] Stern DT, Ben-David MF, De Champlain A, Hodges B, Wojtczak A, Schwarz MR (2005). Ensuring global standards for medical graduates: a pilot study of international standard-setting. Med Teach.

[11] Alkhenizan A, Shaw C (2012). The attitude of health care professionals towards accreditation: a systematic review of the literature. J Family Community Med.

[12] Baker SS, Morrone AS, Gable KE (2004). Allied health dean’s and program directors’ perspectives of specialized accreditation effectiveness and reform. J Allied Health.

[13] Welsh JF, Metcalf J (2003). Cultivating faculty support for institutional effectiveness activities: benchmarking best practices. Assess Eval High Educ.

[14] Azila NM, Tan CP (2005). Accreditation of medical schools: the question of purpose and outcomes. Med J Malaysia.

[15] Simpson I, Lockyer T, Walters T (2005). Accreditation of medical training in Australia and New Zealand. Med J Malaysia.

[16] Hasan T (2010). Doctors or technicians: assessing quality of medical education. Adv Med Educ Pract.

